# Immunoconjugates as an Efficient Platform for Drug Delivery: A Resurgence of Natural Products in Targeted Antitumor Therapy

**DOI:** 10.3390/ph17121701

**Published:** 2024-12-17

**Authors:** Rositsa Mihaylova, Denitsa Momekova, Viktoria Elincheva, Georgi Momekov

**Affiliations:** 1 Department “Pharmacology, Pharmacotherapy and Toxicology”, Faculty of Pharmacy, Medical University of Sofia, 1000 Sofia, Bulgaria; viktoriaelincheva@gmail.com (V.E.); gmomekov@pharmfac.mu-sofia.bg (G.M.); 2 Department “Pharmaceutical Technology and Biopharmaceutics”, Faculty of Pharmacy, Medical University of Sofia, 1000 Sofia, Bulgaria; dmomekova@pharmfac.mu-sofia.bg

**Keywords:** ADC, immunotoxins, targeted antitumor therapy, drug delivery, dolastatins, calicheamicins, maytansinoids, camptothecins, amatoxins, pan-B-cell antigens, HER2, TROP-2, tissue factor, C-MET, Nectin-4

## Abstract

The present review provides a detailed and comprehensive discussion on antibody–drug conjugates (ADCs) as an evolving new modality in the current therapeutic landscape of malignant diseases. The principle concepts of targeted delivery of highly toxic agents forsaken as stand-alone drugs are examined in detail, along with the biochemical and technological tools for their successful implementation. An extensive analysis of ADCs’ major components is conducted in parallel with their function and impact on the stability, efficacy, safety, and resistance profiles of the immunoconjugates. The scope of the article covers the major classes of currently validated natural compounds used as payloads, with an emphasis on their structural and mechanistic features, natural origin, and distribution. Future perspectives in ADCs’ design are thoroughly explored, addressing their inherent or emerging challenges and limitations. The survey also provides a comprehensive overview of the molecular rationale for active tumor targeting of ADC-based platforms, exploring the cellular biology and clinical relevance of validated tumor markers used as a “homing” mechanism in both hematological and solid tumor malignancies.

## 1. Introduction

The achievement of cytotoxicity with maximal selectivity against tumor cells and minimal damage to normal tissues is a canonical principle of anticancer chemotherapy. It was first integrated into Paul Ehrlich’s concept of targeted therapy and subsequently successfully implemented into modern drug development. The identification of key molecular determinants in different cancer types, as well as recent advances made in molecular biology and biopharmaceutical sciences, expedited the development of highly specific immunotherapy drugs and ushered in a new era in personalized medicine. Cutting-edge treatment approaches such as antibody–drug conjugates (ADCs) rely on the active targeting of extremely toxic natural products to tumor cells with specific antigen markers. By design, immunoconjugates combine the potent cytotoxic properties of otherwise inapplicable drugs with the high affinity of the monoclonal antibody toward a specific tumor epitope. As a result, the therapeutic index of the payload agent is dramatically increased, breathing new life into old non-prospective molecules with pronounced off-target toxicities. Targeted therapies, on the other hand, are highly selective towards malignant cells but incapable of eliciting potent cytotoxic effects. The limitations of existing therapeutic modalities have driven the development of hybrid immune-based drugs, combining “the best of two worlds”. The number of drug conjugates in the modern instrumentarium of targeted immunotherapy is growing steadily, and the present review provides insight into clinically approved entities both in the context of their structure and targeted tumor pathologies.

## 2. Design and Structural Characteristics of ADCs

**Figure 1:**
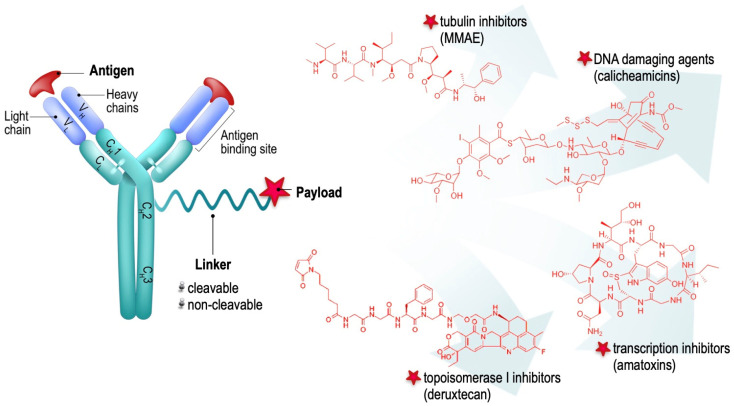
Conceptual structure and major components of ADC-based platforms and validated classes of payloads used in ADC design.

Antibody–drug conjugates (ADCs) are composed of three key components: (1) an antibody that specifically targets an antigen, typically found on the surface of cancer cells; (2) a highly potent cytotoxic drug used as a payload; and (3) a chemical linker serving as a bridging moiety between the drug and the immunoglobulin molecule [1,2,3,4,5,6] (Figure 1). Although governed by a simple concept, the design and development of a functionally active ADC construct is a complex process and requires proper selection of the targeted tumor epitope, the antibody component, its payload, and its bridging domain [7].

### 2.1. The Monoclonal Antibody: “Aiming the Gun”

In ADCs, the antibody is designed to specifically target a tumor-associated antigen that is minimally expressed on normal cells. To reduce immunogenic side effects, early ADCs with murine or chimeric antibodies have been replaced by next-generation humanized or fully human immunoglobulins. Their type and class are also of crucial importance as the main determinants of the plasma circulation time, immunogenicity, modulatory activity on the immune system, and target specificity of the drug [2,3,8,9,10,11]. The class of antibody and its functional properties are determined by the constant “stem” region in the Y-shaped immunoglobulin structure, known as the “crystallization fragment” or Fc. There are five classes of antibodies—IgG, IgM, IgD, IgE, and IgA—eliciting different effector mechanisms for antigen recognition and elimination [12,13]. In addition, the Fc region can interact with special Fc receptors on phagocytes and mast cells, as well as components of the complement system (such as C1q), and mediate the intrinsic effector humoral functions of mAbs, such as antibody-dependent cellular cytotoxicity (ADCC), antibody-dependent cellular phagocytosis (ADCP), and complement-dependent cytotoxicity (CDC) [14,15,16,17]. Endosomal FcRn receptors for IgG are largely responsible for the recycling and the long circulating half-life of this antibody class. In the IgG glycoprotein, the amino acid arginine 297 (N297) in the constant CH2 domain contains a conserved glycosylation site that is essential for the structural conformation of the Fc fragment and its interaction with Fc receptors and the complement factor C1q [18]. The affinity of different IgG subclasses for the Fc receptor decreases in the order IgG1 > IgG3 > IgG4 > IgG2, and thus, the majority of mAb-based antitumor therapies use IgG1 as a scaffold to ensure their long circulating half-life and recruit the potent accessory role of the immune system in battling cancer cells [2,13,19].

Equipping the antibody with a unique recognition surface that is complementary to specific tumor epitopes is also critical for ensuring ADCs’ high affinity and effective target delivery in malignant cells [20]. Ideally, the molecular target should be abundantly expressed on the surface of cancerous cells while showing minimal or no expression in normal tissues [8,21]. Systematic exploration and mapping of the molecular landscape in some of the most common malignancies revealed suitable for targeting tumor markers in both hematological and solid neoplasms [22]. For example, the HER2 and the trophoblast cell surface 2 (TROP2) antigens are currently utilized in ADC development for breast cancer and other epithelial malignancies exhibiting abnormal expression, whereas the so-called pan-B-cell antigens serve as feasible anchors for active tumor targeting in non-Hodgkin lymphoproliferative diseases [23,24,25].

To overcome the challenge of insufficient internalization, Li et al. (2016) developed a bispecific antibody aimed at two distinct epitopes of the HER2 antigen, enhancing binding affinity to the therapeutic target on tumor cells [26]. For example, an anti-HER2 biparatopic antibody exhibited advantageous internalization, lysosomal trafficking, and stability of the antibody–antigen complex compared to the traditional monofunctional immunoconjugate Trastuzumab emtansine and induced superior T-cell activation [26,27,28,29]. Bispecific ADCs can also be designed to simultaneously target two heterogeneous epitopes with overlapping functions in tumor pathology [30,31]. Trifunctional antibodies have also been exploited in ADC development, providing further augmentation of immune-mediated cytotoxicity via the costimulatory CD28 T-cell signaling and the executor natural killer (NK) antitumor response [30,32]. However, the increased binding affinity of polyfunctional ADCs raises concerns about their potential participation in cross-reactivity and on-target toxicity in healthy tissues, and selecting the right combination of molecular targets remains a significant challenge.

### 2.2. The Cytotoxic Payload: “The Bullet Drug”

The payload is the highly toxic effector molecule intended to kill target cells upon its internalization and release [29]. Due to their high toxicity, these molecules require precise tumor delivery by the monoclonal antibody, serving as both a carrier and a navigation tool to minimize off-target damage to healthy cells. The ideal ADC payloads should meet several key characteristics [4,33].

- Extremely high cytotoxicity (reaching 0.001–1 nM): due to the limited availability of tumor-specific antigens, especially in solid tumors, and the relatively low internalization efficiency of monoclonal antibodies, the payload must exert potent anticancer activity at low picomolar concentrations [33,34];
- Low Immunogenicity: Minimizing immunogenicity is essential to avoid compromising the ADC’s efficacy or causing severe side effects. This can be achieved by using highly toxic non-protein compounds derived from plants, animals, or microorganisms or by opting for smaller molecular payloads [28,33,35].
- High Stability: Given the long plasma half-life of antibodies, ADCs must remain stable in circulation to prevent premature release of the payload and systematic exposure to it. The cargo molecules should also be stable within the cytoplasm and lysosome, resisting degradation under low pH conditions [33,36];
- Modifiability: Payloads should have functional groups that allow modification and linker attachment without reducing their potency. These groups must be carefully chosen to maintain the drug’s efficacy, particularly when using non-cleavable linkers, where the payload must retain its potency post-antibody degradation [33,37];
- Bystander Killing Effect: ADCs with highly permeable and diffusible payload molecules can induce a bystander killing effect, affecting adjacent tumor cell populations with low or absent target antigen expression. This neighboring effect is particularly valuable in tumors with borderline or heterogeneous antigen expressions (such as the HER2-low breast cancer phenotype), bypassing the prerequisite for antigen-dependent internalization of the toxic drug [38]. However, this phenomenon must be carefully balanced, as highly permeable, hydrophobic payloads can also increase off-target toxicity by affecting healthy tissues [33,39].
- Water Solubility: Suitable water solubility is crucial to ensure that the payload can be conjugated to the antibody and that the resulting ADC construct remains stable and soluble under physiological conditions. Excessive hydrophobicity can further lead to aggregation and instability, negatively affecting both ADC stability and the bystander killing effect [2,33,34].
- Intracellular Targeting: Since most ADCs are designed to deliver their payloads inside tumor cells, the cargo drugs should be aimed at sensitive intracellular components of the cell division machinery. Agents that primarily act on membrane structures, such as ion channels or clotting factors, are generally unsuitable for ADCs [33,40].

Besides the physicochemical and mechanistic features of the payload, ADC performance is also influenced by its loading capacity (the number of loaded molecules or the drug–antibody ratio, DAR), as well as the presence of multidrug resistance (MDR) efflux pumps in the malignant tumor that may limit drug accumulation at the site of action [27]. While packing the carrier antibody with multiple effector molecules has a boosting effect on ADC’s potency, it raises other stability and safety concerns, including protein aggregation and off-target toxicity, that need to be carefully weighed [29].

#### 2.2.1. Naturally Occurring Toxins Used as Payload in Immunoconjugates

**Figure 2:**
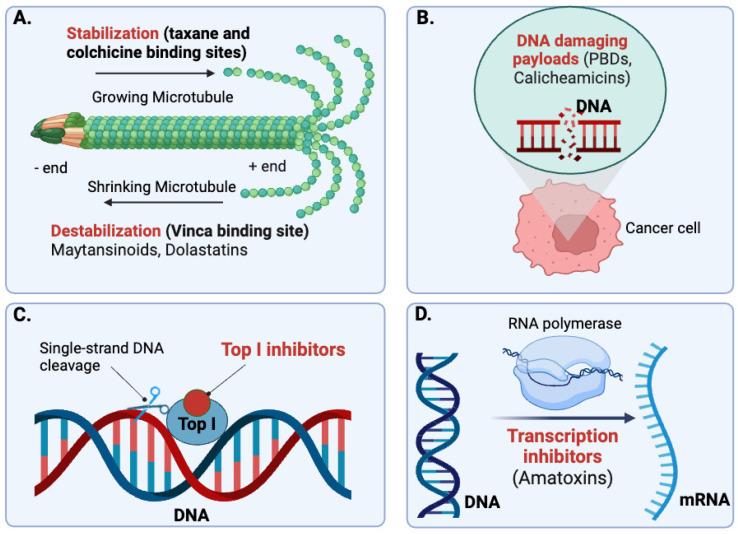
Mechanism of action of the major classes of naturally occurring toxins used as payloads in ADC design: (**A**) Antimitotic agents, (**B**) DNA-damaging agents, (**C**) Topoisomerase I inhibitors, and (**D**) Transcription inhibitors. Created in BioRender. Mihaylova, R. (2024).

Depending on their cellular targets and primary mechanism of cytotoxicity, payload agents in ADCs are generally classified into four main categories: tubulin inhibitors, DNA-damaging agents, topoisomerase I inhibitors, and transcription inhibitors (Figure 2) [2]. Most of these agents are naturally occurring toxins with potencies several orders of magnitude higher than conventional cytostatics, depriving them of a therapeutic window as free drugs [36].

##### Antimitotic Agents

Tubulin inhibitors are potent and highly toxic payload molecules that induce cell cycle arrest and apoptosis by disrupting microtubule dynamics within cancer cells. With the exception of the synthetic auristatins (MMAE, MMAF), most of these compounds are of natural origin, including maytansinoids (DM1, DM4) and dolastatins.

**Maytansinoids**

**Figure 3:**
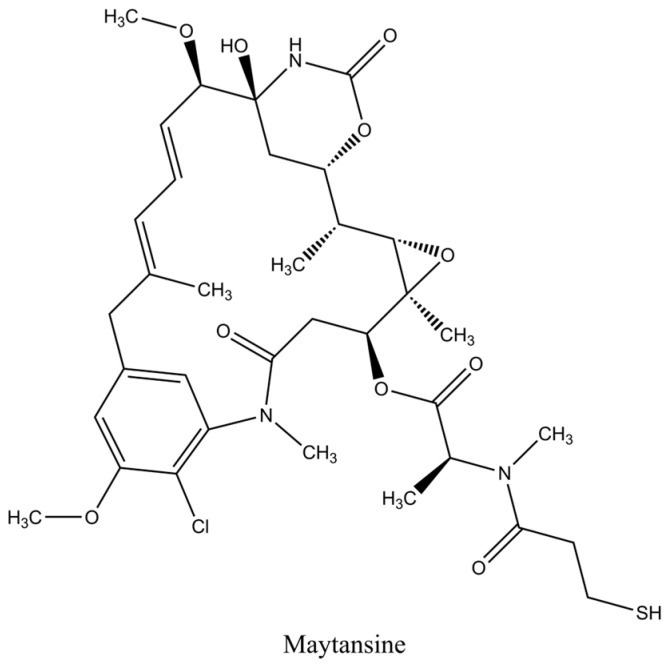
Chemical structure of maytansine I.

Maytansinoids are a class of cytotoxins with structural similarities to rifamycin, geldanamycin, and ansatrienin. The prototypical compound maytansine I is the most active member of the group, first isolated from the Ethiopian shrub Maytenus ovatus [41]. This ansa macrolide is a 19-member lactam featuring a chlorinated benzene ring chromophore and various functional groups, including carbinolamine, epoxide, or aryl (Figure 3) [42]. Over the years, several maytansine derivatives have been isolated from natural sources, including bacteria (Actinosynnema pretiosum), mosses, and higher plants (Colubrina texensis and Trewia nudiflora), with differences primarily in the ester side chain at the C-3 position [43]. Maytansine and its derivatives are potent inhibitors of microtubule assembly, binding to tubulin’s β-subunit at or near the vinblastine-binding site [44]. In studying the structure−activity relationships of maytansinoids, the lactam moiety has been identified as the pharmacophore unit, leaving the terminal N-acyl group in the side chain available for chemical modification [33]. The consequent defects in microtubule organization induce mitotic arrest at the G2/M cycle of treated cells, producing effects similar to those of vinblastine [36,45]. The antimitotic activity of maytansine I is about 1000 times higher than that of Vinca alkaloids, but its clinical development has been discontinued due to lack of tumor selectivity and intolerable adverse reactions [46]. However, semi-synthetic analogs of maytansine with a terminal thiol group (i.e., DM1, DM4) suitable for disulfide conjugation were derived and became among the first payload families to be successfully used in commercially available ADCs such as Trastuzumab emtansine (FDA, EMA 2013) [33,47].

**Dolastatins**

**Figure 4:**
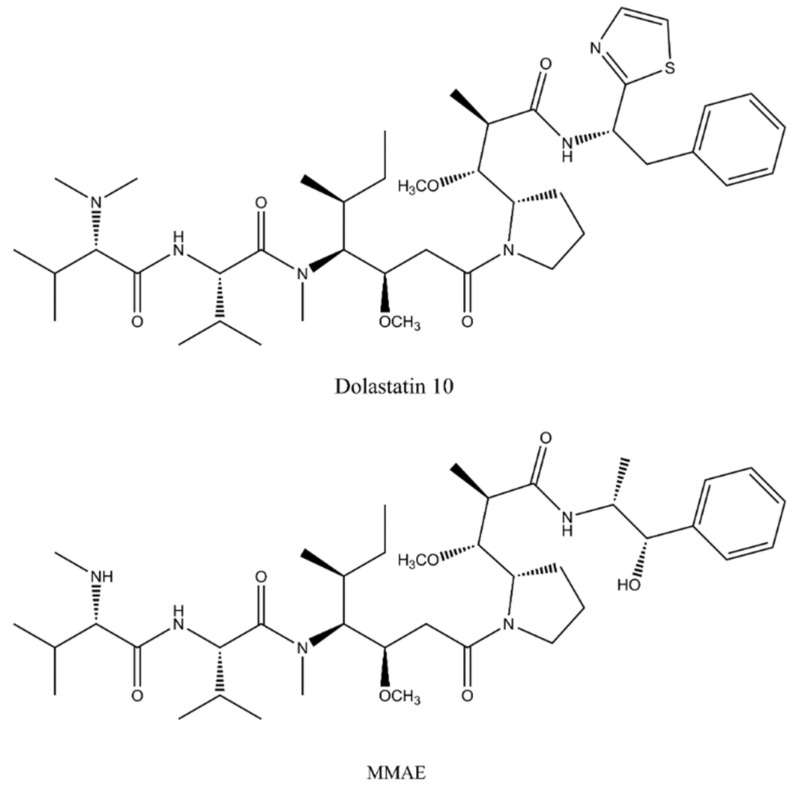
Chemical structures of the prototype dolastatin 10 and its synthetic analog monomethyl auristatin E (MMAE), used as a microtubule-stabilizing drug in ADCs.

In the mid-1960s, Pettit and colleagues initiated systematic research on marine organisms to identify novel anticancer agents. By the late 1980s, this research had resulted in the identification of a new class of natural compounds with potent cytotoxic activity, named dolastatins after their original source—the marine biota Dolabella auricularia inhabiting the Indian Ocean [36,48]. The dolastatin family represents a number of linear and cyclic short-chain pseudopeptides (dolastatins 1–18) composed of unusual amino acid residues. Of these, dolastatin 10 was identified as the most promising lead compound, demonstrating picomolar cytostatic activity in both in vitro and in vivo models of non-Hodgkin lymphoma, melanoma, small cell lung carcinoma, human prostate cancer, among others [49,50]. Although regarded as a peptide, elucidation of its chemical structure discovered four actual amino acid residues, namely (S)-dolavaline, (S)-valine, (3R,4S,5S)-dolaisoleuine, and (2R,3R,4S)-dolaproine, while (S)-dolaphenine is an unusual C-terminal extension of a peculiar primary amine possibly stemmed from phenylalanine [50,51]. Detailed mechanistic studies revealed that dolastatin derivatives inhibit tubulin polymerization by targeting a “peptide site” close to the vinca alkaloid binding domain and, similar to maytansinoids, induce metaphase arrest in tumor cells. However, due to the occurrence of pronounced neutropenia and peripheral neuropathy common to antimitotic agents, clinical investigation of the prototype member dolastatin 10 has been discontinued [50]. Successfully translated into clinical practice, on the other hand, are a couple of synthetic dolastatin analogs with improved properties and water solubility, namely monomethyl auristatin E (MMAE) and monomethyl auristatin F (MMAF), used as payload molecules in several commercially available ADCs (Figure 4) [49].

##### DNA-Damaging Agents

The idea of selectively inducing DNA damage in tumor cells was conceptualized in the very early ADC constructs. However, the first attempts using doxorubicin as a payload molecule (BR96-DOX) proved wildly discouraging, paradoxically due to the insufficient potency of the anthracycline limited in the nanomolar range [52]. Since then, a number of extremely toxic DNA alkylating (pyrrolobenzodiazepine, PBD) or fragmenting (calicheamicins) agents have been discovered and successfully used as payloads in modern, high-performance ADCs (Tables 2 and 3) [27,53,54].

**Calicheamicins**

Calicheamicins were first explored in the mid-1980s by Lederle Laboratories (Pearl river, NY, USA) during their search for new fermentation-derived antitumor antibiotics [36]. These compounds were isolated from Micromonospora echinospora sp. Calichensis and bear structural similarities with esperamycins and dynemicins (Micromonospora chersina), comprising a unique 10-member macrocyclic structure with a conjugated system of at least one double (“en”) and two triple (“di-yne”) bonds [55]. This peculiar structure renders them a new class of extremely toxic antibiotics named “enediynes”, which in their natural environment are stabilized in a chromoprotein complex, shielding the producing microorganisms from their drastic effects [56,57].

**Figure 5:**
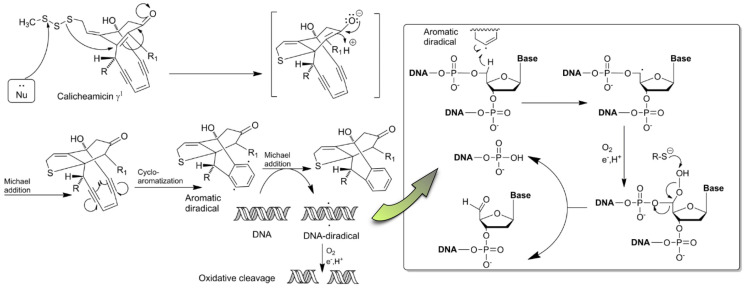
Structure and mechanism of activation of calicheamicin-γ. The process is initiated by a nucleophilic attack in vivo (presumably by glutathione) directed to the trisulfide group, with subsequent intramolecular Michael addition to the reactive α,β-unsaturated ketone. The resulting intermediate spontaneously cycloaromatizes to form aromatic diradical species, abstracting two hydrogen atoms from DNA’s counter-deoxyribose sugars. This transforms the nucleic acid into a highly reactive diradical that, in turn, reacts with O_2_, leading to extreme levels (>95%) of DNA fragmentation [59,60].

As with many other DNA targeting agents, enediynes are inactive in their primary state. A spontaneous or nucleophile-driven cycloaromatization enables the formation of highly reactive 1,4-benzenoid diradical species, attacking and abstracting two hydrogen atoms from the sugar opposite strands at the minor groove of the DNA helix. This, in turn, transforms DNA into a diradical, causing the formation of interstrand crosslinks and subsequently inducing double- or single-stranded DNA ruptures (Figure 5) [56,58,59].

Enediynes exhibit phenomenal cytotoxicity at femtomolar concentrations, exceeding thousands of times that of conventional cytostatics, which limits their use as therapeutic agents in their natural free form [60]. However, several polymer-based delivery systems and immunoconjugates with the prototype calicheamicin-γ have been exploited and shown great clinical promise as anticancer treatments [61]. The first commercially available ADC with an enediyne antibiotic payload was gentuzumab ozogamicin, targeting the CD33 antigen on the surface of myeloid leukemia cells and indicated for the treatment of elderly patients with relapsed AML [62]. Shortly after, in 2017, an anti-CD22 monoclonal antibody carrying the same payload, lnotuzumab ozogamicin, received approval for relapsed or refractory B-cell precursor leukemia treatment [63].

**Pyrrolo[1,4]benzodiazepines**

Anthramycin, sibiromycin, and tomaymycin are naturally occurring antibiotics of the pyrrolo[1,4]benzodiazepine (PBD) family that selectively target DNA’s minor groove to form covalently linked adducts. Their extraordinary anticancer activity is mediated by the generation of highly reactive imine intermediates that alkylate the C2 amino group of purine bases. Despite exhibiting promising inhibitory activity in various malignant tumors, these compounds have been associated with significant cardiotoxicity and the onset of tissue necrosis, limiting their potential for development as clinically administered drugs [64,65].

**Figure 6:**
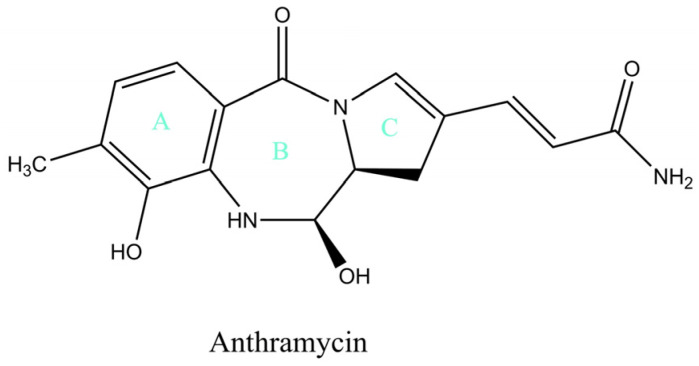
Chemical structure of anthramycin.

Of the PBD family, anthramycin (isolated from Streptomyces refuineus var. thermotolerans) was the first antibiotic discovered and studied for its potent antitumor properties. Since the 1960s, many natural derivatives carrying the same tricyclic structure of an aromatic A-ring, a 1-4-diazepin-5-one B-ring, and a pyrrolidine C-ring have been investigated (Figure 6), and, more recently, rationally designed synthetic PBD dimers have emerged as a new class of feasible payloads for antibody–drug conjugates [53,66]. Similar to alkylating agents, natural pyrrolobenzodiazepines exert monofunctional DNA cross-linking by covalently binding the exocyclic C2 amino groups of guanines, spanning over three DNA base pairs and perfectly fitting within the DNA minor groove [67]. Synthetically derived PBD dimers, on the other hand, carry two PBD molecules joined through their C8 positions via a flexible tether and can, therefore, produce alternative bifunctional DNA cross-links with greater resistance to DNA repair mechanisms [53,68,69]. Given their significantly higher potency and alternative mode of DNA targeting, PBD dimers have been widely exploited as ADC warheads, and the first immunoconjugate of this design, Loncastuximab tesirine, is now in clinical use for the treatment of relapsed/refractory diffuse large B-cell lymphoma [53].

##### Topoisomerase I Inhibitors

Topoisomerase I (Top I) is a ubiquitous and essential eukaryotic enzyme required for the relaxation of DNA supercoiling generated in the replication complex [70]. Given the higher growth rate and continuous active division of cancerous cells, Top I is much needed and, therefore, highly expressed by malignant tumor populations, making it a convenient target for pharmacological inhibition. Accordingly, Top I inhibitors exert a certain selectivity in their antitumor action by preferentially disrupting DNA replication in malignant cells, leading to apoptotic death [22]. The first class of these drugs to be identified and introduced into clinical practice were derivatives of the camptothecin alkaloid, isolated from the bark of the Chinese ornamental tree Campotheca acuminata as the result of a large-scale screening program of the US National Cancer Institute in 1966 [71].

Camptothecin comprises a planar pentacyclic ring system that is essential for its biological activity. It includes a pyrrolo[3,4-β]-quinoline moiety (rings A, B, and C), a conjugated pyridone (D), and an α-hydroxy lactone ring (E) with a chiral center at position C20 occupying (S) configuration [72,73,74]. Due to its high toxicity and low water solubility, the prototypical natural compound did not succeed clinically as a cytostatic agent, which prompted the development of its semi-synthetic derivatives (topotecan and irinothecan) through structural modifications allowing increased water solubility and stabilization of the lactone form [71].

Top I is the only cellular target of camptothecins, and initial studies elucidated their unique mechanism of interfacial enzyme inhibition. It appears that Top I in its resting state is particularly resistant to inhibition but, during the formation of the cleavage complex, is prone to entrapment in a ternary complex, with the drug molecule being stacked between the base pairs at the DNA cleavage site, “clamped” by the active TOP1ccs. Thus, the drug simultaneously binds both TOP1ccs via a network of hydrogen bonds and the DNA matrix via hydrophobic interactions. Moreover, crystallographic analyses with the topotecan derivative indicate that binding is stereo-specific and can only occur for the active 20-S enantiomer of the camptothecin molecule [70,75,76,77].

Camptothecin derivatives exhibit a potent anticancer activity in the micromolar to nanomolar range, particularly against solid tumor malignancies of epithelial origin, including ovarian and lung carcinomas and colorectal cancer [78,79]. The semi-synthetic analog irinotecan has been shown to serve as a prodrug whose urethane functional group undergoes enzymatic hydrolysis in vivo to the active metabolite SN-38 (7-ethyl-10-hydroxycamptothecin). SN-38 is about 1000 times more cytotoxic than the parent compound, reaching picomolar activity [80]. However, only 2–5% of the administered dose is converted to the active metabolite, which drove interest in the development of its drug delivery systems, including immunoconjugates [81]. Indeed, several ADCs of SN-38 have shown superior efficacy and safety in oncological clinical trials, alleviating the dose-limiting neutropenia and gastrointestinal toxicities of irinotecan [82,83]. In 2020, Sacituzumab govitecan received accelerated FDA approval for the treatment of metastatic triple-negative breast carcinoma (mTNBC) based on clinical results from the large-scale Phase 1/2 study IMMU-132-01 evaluating its efficacy in epithelial cancers. Full approval was granted in 2023, and the drug is currently under review for marketing authorization for the treatment of metastatic urothelial carcinoma [84,85,86].

**Figure 7:**
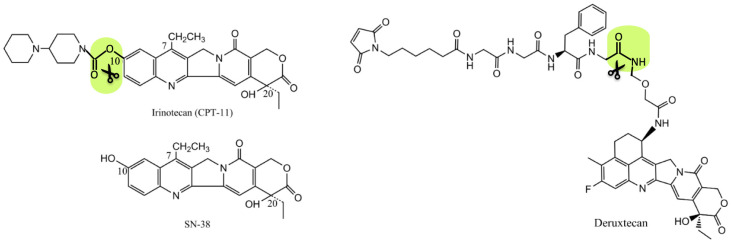
Chemical structures of camptothecin derivatives: Irinotecan undergoes enzymatic conversion by carboxylesterases to its active metabolite SN-38. Deruxtecan carries a tetrapeptide Gly-Gly-Phe-Gly linker, cleaved by lysosomal cathepsins.

In addition, an ADC with a novel HER2-targeted exatecan derivative (Fam-trastuzumab deruxtecan) was developed and licensed in the US as a second- or later-line treatment of several epithelial solid tumor malignancies with abnormal HER2 signaling, including breast, stomach cancers, and non-small cell lung carcinoma [87,88]. More importantly, based on the DESTINY-Breast04 study, trastuzumab deruxtecan was the first immunoconjugate to be approved as a treatment option for the newly defined HER2-low breast cancer subtype due to its pronounced bystander killing effect, high DAR (up to eight carried molecules), and the fine-tuned drug release kinetics by an enzymatically cleavable tetrapeptide linker (Figure 7) [89,90].

##### Transcription Inhibitors

**Amatoxins**

Transcription inhibitors such as amatoxins represent a novel approach to the development of antibody–drug conjugates (ADCs). Amatoxins are a group of highly toxic cyclopeptides derived from basidiomycetes mushrooms, particularly those belonging to the Amanita genus [91]. The green death cap mushroom (Amanita phalloides) is a prominent source of amatoxins and has historically been associated with fatalities, causing 95% of all fatal mushroom poisonings globally. Amatoxins, when ingested, are preferentially taken up by liver cells via the organic anion transporting polypeptide 1B3 (OATP1B3), leading to acute liver injury and subsequent organ failure. This organ-targeted toxicity historically precluded their clinical investigation and medical use as stand-alone antitumor agents [36,92,93].

**Figure 8:**
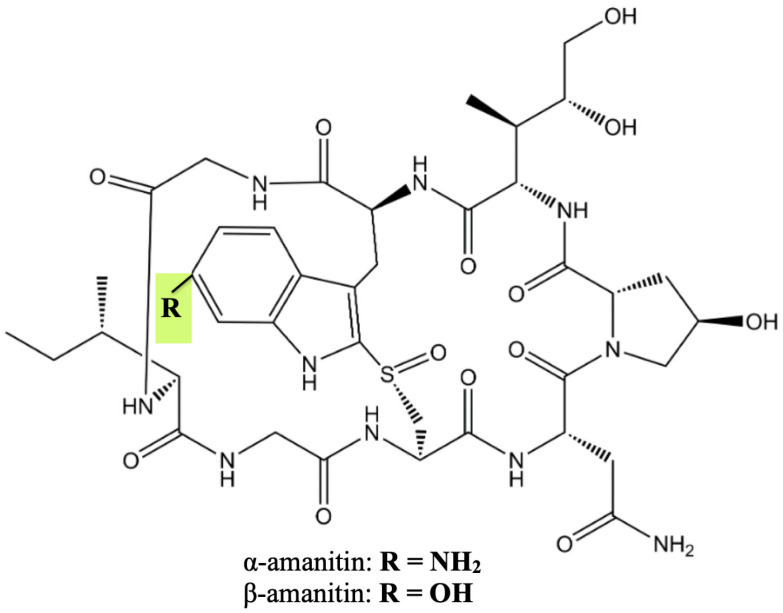
Chemical structures of α- and β-amanitin.

The amatoxin structure features a bicyclic ring system of eight L-configurated amino acids with an intramolecular sulfoxide bridge between tryptophan and cysteine residues, forming an outer and inner loop (Figure 8). The presence of three hydroxylated side chains significantly enhances their water solubility and contributes to their binding affinity to the targeted RNA polymerase II enzyme [92]. Notably, regardless of their peptide structure, amatoxins exhibit great heat, acid, and enzyme stability, making them resistant to degradation by thermal processing and gastrointestinal tract conditions [94,95]. α-amanitin and β-amanitin (differing by the presence of an NH2 and OH group, respectively) are the most prevalent and potent representatives of the family, comprising about 90% of the total amatoxins [96].

In 1966, Stirpe and Fiume demonstrated that α-amanitin exerts robust inhibition of RNA synthesis in isolated mouse liver nuclei sufficient to ultimately cause protein depletion and cell death [97]. Further investigations revealed that the primary mechanism of amatoxins’ action is inhibition of RNA polymerase II, targeted by non-covalent binding, particularly in its bridge helix region and the catalytic trigger loop segment, serving for substrate recognition and elongation of the mRNA transcripts [94].

The hydrophilic properties of amatoxins offer distinct advantages for their use as warheads in ADCs. Firstly, their structure provides convenient functional groups for linker attachment and high aqueous solubility, which facilitate the conjugation process. Additionally, amatoxins are less likely to cause ADC aggregation, an issue often observed with hydrophobic payloads. Furthermore, the low molecular weight and strong hydrophilicity of the released toxin ensure their rapid renal clearance, reducing the risk of off-target accumulation in healthy tissues [36,95]. It is also noteworthy that their poor substrate affinity for the multidrug resistance (MDR1) efflux transporter accounts for the high efficacy of amanitin-based ADCs in MDR-overexpressing tumors [95]. Currently, several immunoconjugates of this class are under clinical development and on the verge of marketing authorization, the most advanced of which is HDP-101—a B-cell maturation antigen (BCMA)-targeted ADC showing great promise for the treatment of relapsed/refractory multiple myeloma [33].

##### Other Naturally Occurring Toxins as Payload Candidates

A renewed trend in ADC design is the employment of highly potent toxins naturally produced by plants, fungi, and bacteria as part of their defense systems against pathogenic microorganisms, viruses, and insects, particularly under stressful conditions. Many of these toxins belong to the ribosome-inactivating proteins (RIPs) family, displaying a wide array of biological activities, including antiproliferative, antitumor, immunomodulatory, antiviral, antifungal, and insecticidal effects [98,99]. Type I RIPs are monomeric single-chain peptides, whereas type II exist in a heterodimeric form, featuring A and B polypeptide chains linked via a disulfide bond. Both classes of RIPs block protein translation as a result of the rRNA N-β-glycosylase activity of the catalytic A chain, which irreversibly cleaves a single adenine base at a conserved site of the 28 rRNA subunit and interferes with the binding of elongation factors needed for transpeptidation [99,100,101]. Beyond protein synthesis, some RIPs have been reported to exert pleiotropic enzymatic activities, including depurinating activity on non-ribosomal RNA and DNA [100]. However, the cytotoxic potential of the more common type 1 RIPs is significantly lower, given the absence of the lectin-like B chain required for attachment and galactosyl-specific internalization of the toxin into host cells [101,102,103]. Notably toxic type II RIPs include ricin (*Ricinus communis*), abrin (*Abrus precatorius*), volkensin (*Adenia volkensii*), and modeccin (*Adenia digitata*). Type I RIPs are predominantly found in seeds, but also in leaves and roots, across plants in Asteridae, Caryophyllidae, Liliidae, Magnoliidae, and Rosidae subclasses, with the highest diversity in Cucurbitaceae, Euphorbiaceae, and Fabaceae families [36,98,104]. Among the vast realm of plant-derived type I RIPs, only five have been clinically evaluated as conjugated agents, mostly for the treatment of lymphoproliferative diseases: saporin (*Saponaria officianalis*), pokeweed antiviral protein (PAP) (*Phytolacca americana*), gelonin (*Gelonium multiflorum*), momordin (*Momordica balsamina*), and bouganin (*Bouggainvillea spectavilis*) [105].

Oddly, for immunotherapy purposes, the more potent type 2 holotoxins are non-viable candidates, given their facile uptake by all host cells, precluding the possibility of selective tumor delivery; therefore, only bare A chains derived from type II or type I RIPs have been explored as payload molecules for monoclonal antibodies or other carriers (i.e., lectins, hormones, growth factors, etc.) to form immunotoxins (ITs) or other protein-based therapeutics with active targeting properties [99,106,107]. Research into immunotoxins as anticancer agents has been extensive, driven by evidence that the introduction of a single toxin molecule into the cytosol is sufficient to induce cell death [108]. Moreover, advances in transcriptional targeting, linkage type, and novel fusion techniques have provided opportunities for improving cellular internalization, intracellular processing, and routing of the IT conjugate in malignant cells [100,109]. Additionally, the presence of distinctive antigenic and active site residues in RIPs has enabled the development of RIPs with preserved biological activity but reduced immunogenicity, which has been a major hurdle in IT development [98,110].

Among bacterial single-chain RIPs, the pseudomonas exotoxin A (PE) and the diphtheria toxin (DT) have emerged as payload agents in a variety of carriers, targeting eukaryotic translation through the NAD-dependent ADP-ribosylation of the elongation factor-2 [107,111,112]. Indeed, several ITs with native or recombinant forms of these toxins have been developed and progressed to clinical trials, of which the recombinant PE38 version of PE has proven most successful. Moxetumomab pasudotox, a CD22-directed antibody fragment conjugated to a truncated 38kD version of Pseudomonas exotoxin A (PE38), received FDA approval (Lumoxiti^®^) in 2018 for the treatment of adult patients with relapsed/refractory hairy cell leukemia (HCL) but was permanently withdrawn from the market due to reasons not related to safety or efficacy [113,114].

### 2.3. The Linker: “Pulling the Trigger”

The linker is the spacer component that chemically binds the cytotoxic payload to the antibody. It is designed to remain stable in the bloodstream, keeping the drug from premature discharge and only releasing the payload upon internalization and intracellular processing in the target cells [11]. The characteristics of the linker, such as being cleavable or non-cleavable, steric hindrance, length, and release kinetics, can also significantly impact the toxicity, solubility, and stability of the ADC [29,115,116]. A properly selected and suitable linker allows the achievement of 100 to 1000 times higher therapeutic concentrations at the site of action compared to conventional treatments, reducing overall toxicity [117]. The method and site of conjugation determines the number of payload molecules that can be attached to the antibody. The drug–antibody ratios (DARs) of ADCs range from 2 to 8, and the final product is often a mixture of ADCs with varying DAR numbers [118]. To reduce the heterogeneity in drug–antibody ratios, genetic engineering can be used to introduce site-specific conjugation sites in the third-generation ADC constructs, providing tighter control of the number of drugs conjugated per antibody [119,120,121]. The advances made in the field of bioorthogonal chemistry and protein engineering offer state-of-the-art techniques for fine-tuning the stoichiometry and site of conjugation in next-generation ADCs, yielding more homogeneous conjugates. THIOMAB™ antibodies bearing engineered cysteine residues have displayed improved in vivo stability, longer circulating half-live, and larger therapeutic indexes in preclinical studies. Encouraging results have also been achieved using site-specific conjugation of native antibodies using engineered microbial transglutaminases and the recently reported traceless site-selective conjugation AJICAP-M method. Using Fc-affinity peptides, AJICAP-M enables cysteine-specific conjugation of a broad range of payload molecules and antibodies with varying affinities and specificities, producing ADCs with greater homogeneity and reproducibility [122,123,124,125,126,127].

Cleavable linkers further differ in their mechanism of deconjugation, as they may release their effector cytotoxic payload via chemical reduction, enzyme-mediated hydrolysis, or in response to pH change. In the first case, the drug molecule and the antibody are usually bridged by a disulfide bond, and the “magic bullet” is released by reducing agents that are abundant in the tumor microenvironment (such as glutathione) [11,116,120]. Enzymatically cleavable linkers may be cathepsin B, β-glucuronidase, or phosphate sensitive, whereas hydrazone linkers are pH-responsive, exhibiting high stability in the bloodstream (neutral pH 7.4) and preferential release of their cargo in acidic cellular compartments such as the lysosomes (pH < 5) or late endosomes (pH 5.5–6.2) [3,128]. Cleavable linkers are essential to unfold the bystander killing effect of the payload; however, they may favor the diffusion of free toxins into healthy tissues [29]. Non-cleavable linkers (such as SMCC thioether and maleimidocaproyl linkers), on the other hand, require a complete unspecific lysosomal breakdown of the entire ADC scaffold for the payload to be released but provide greater stability in circulation and may contribute to an improved safety profile compared to other conjugation approaches [4,8,27,28,36].

Cathepsin B-labile linkers are one of the most successful conjugation strategies in ADC design, providing improved plasma stability and tumor-selective payload release. Cathepsin B is a lysosomal protease with a relatively broad spectrum of substrate specificity and is overly expressed in many cancer types, especially metastatic tumors [129,130]. Upon internalization and lysosomal transportation of the ADC, the cysteine protease facilitates the cleavage of dipeptide linkers, such as phenylalanine-lysine (Phe-Lys), valine-alanine (Val-Ala) and valine-citrulline (Val-Cit), recognizing the C-terminal side of the sequence. A further optimization of the linker is required when the payload molecule is too bulky, by implementing an additional spacer (i.e., para-aminobenzyl carbamate, PABC) to separate them and prevent aggregation. Phe-Lys-PABC and Val-Cit-PABC are commonly used in ADCs with MMAE, with the latter showing higher stability (half-life of up to 80 h) and reduced off-target toxicity [2,3,4,8,29].

However, conventional Val-Cit linkers may still experience some technological limitations, such as proneness to hydrophobicity-induced aggregation and premature payload release due to off-target enzyme cleavage by carboxylesterases or neutrophil elastases. These shortcomings in performance have been recently addressed by next-generation Exo-cleavable linkers, where the peptide moiety is repositioned at the exo-position of the p-aminobenzylcarbamate spacer. Watanabe et al. report on the design, synthesis, and oncopharmacological evaluation of Exo-cleavable EVC and EEVC linkers attaching MMAE and exatecan to a HER2-targeted Trastuzumab scaffold. Compared to the traditional linear configuration approach, the incorporation of exo-linkers into ADC demonstrated superior in vitro and in vivo activity in HER2+ gastric cancer xenograft models and increased DARs and resistance to aggregation and erratic enzyme-driven release [131,132].

## 3. Mechanism of Action of ADCs

**Figure 9:**
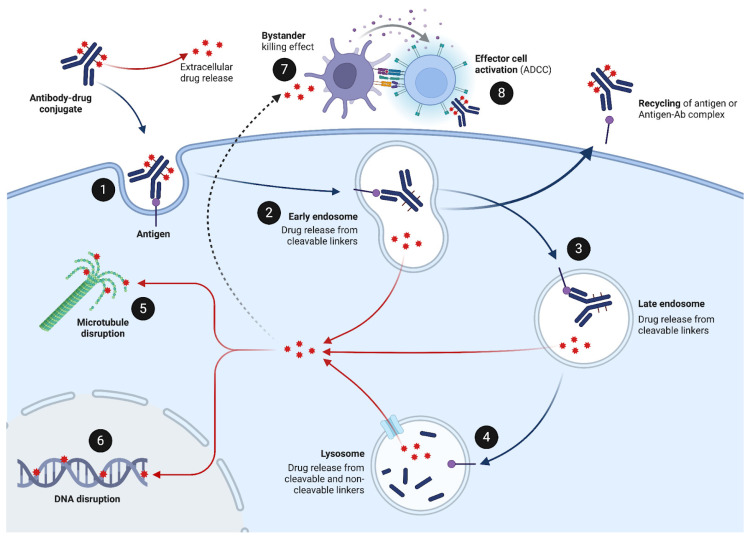
Antibody–drug conjugate drug release and mechanism of action. The monoclonal antibody navigates ADC to a target antigen highly expressed on cancer cells (1) and induces its internalization by the formation of an early endosome (2). The latter matures into a late endosome (3), which then fuses with the lysosome (4). Within the lysosome, lysosomal proteases detach the cytotoxic payload from the antibody, allowing it to target cancer cell components such as microtubules or DNA (5,6). The payload, which is permeable through the membrane, can diffuse back into the extracellular matrix, potentially exerting a bystander killing effect on neighboring cells with low or no antigen expression (7). Additionally, cross-binding of the targeted mAb to the Fc receptors (FCRs) on immune cells (natural killer cells and macrophages) recruits immune-mediated antitumor responses, such as antibody-dependent cell-mediated cytotoxicity (ADCC), complement-dependent cytotoxicity (CDC), and antibody-dependent cellular phagocytosis (ADCP), leading to direct cell death (8). Created in BioRender. Mihaylova, R. (2024).

For most ADCs carrying cytotoxic payloads, the process leading to cancer cell death involves several key steps: highly specific complementary binding of the tumor-associated antigen; receptor-mediated internalization of the ADC following the endosome–lysosome pathway; specific or non-specific dissociation of ADC components, separating the payload from the antibody and linker; payload release into cancer cell’s cytoplasm or nucleus; and disruption of DNA or microtubule function, ultimately leading to cell death (Figure 9) [28,29,115]. Additionally, the monoclonal antibody readily inhibits downstream signaling pathways specific to the targeted antigen that often promote cell survival and proliferation. For instance, trastuzumab in T-DM1 binds to the HER2 receptor, blocking its dimerization and co-activation by the HER1, HER3, or HER4 members of the EGFR tyrosine kinase family and its downstream MAPK and PI3K effector phosphokinase pathways [133].

## 4. Suitable Molecular Targets and Clinical Indications of ADCs

### 4.1. Molecular Targets of ADCs in Hematological Malignancies

Hematological neoplasms are among the most common cancers worldwide; however, in recent decades, we have witnessed a decreasing trend in their mortality rate, which is undoubtedly related to the clinical development and commercialization of modern targeted therapies for their management [134]. Since leukemic malignant cells are abundant and readily accessible in circulation, they are generally more susceptible to targeted therapy compared to solid tumors, where drug delivery is challenged by poor vascularization and limited drug penetration [135]. Since ADCs are administered into the bloodstream, they have direct and immediate access to surface-expressed cluster antigens specific to the immune lineage. Advancements in sequencing technologies continue to shape the genetic and molecular landscapes of oncohematological disorders, thus revealing new molecular targets for their treatment, as well as essential biomarkers for diagnosis, prognosis, and assessing treatment response to therapy (Table 1) [134,136]. In addition to being widely expressed on malignant leukemic cells, the antigens targeted by the monoclonal antibodies in ADCs are often absent from hematopoietic stem cells (HSCs) and show limited to no distribution in normal non-hematopoietic tissues, allowing replenishment after temporary depletion by ADCs [22]. Clinically approved ADCs for the treatment of hematological malignancies are summarized in Table 2.

#### 4.1.1. Cellular Differentiation Markers in Lymphoproliferative Diseases

**The Pan-B-Cell Antigens**

CD19, CD20, CD22, and CD79a are phenotypic surface markers expressed on peripheral B-cell populations, making them a fitting approach for specific B-cell targeting, particularly for B-cell non-Hodgkin lymphomas (B-NHLs) and other B-cell-derived leukemias [22,139]. CD19 expression starts upon B-cell lineage commitment and remains until the terminal plasma cell stage. It serves alongside CD21 as a signaling molecule in the B-cell receptor (BCR) complex, playing a key role in adaptive immune responses [138]. CD19’s rapid internalization after ADC binding and lack of shedding from the cell surface into circulation make it a rational target in ADC design [22,133]. However, its cellular uptake is negatively affected by CD21 expression, indicating that anti-CD19 ADCs might be less effective in CD21-high B-cell cancer phenotypes [22]. As mentioned, an anti-CD19 monoclonal antibody, Loncastuximab tesirine, is used for the targeted delivery of its PBD payload into diffuse large B-cell lymphoma cells. In the pivotal LOTIS-2 phase 2 study, Loncastuximab tesirine demonstrated significant therapeutic efficacy as a single agent, achieving a response rate of 48.3% in patients with r/r DLBCL. A follow-up LOTIS-5 clinical trial was launched in 2020 and will continue recruiting into 2028, assessing the synergistic activity of Loncastuximab and Rituximab versus standard monotherapy approaches [141].

Another inherent B-lymphoid marker used as a homing mechanism for several ADCs is CD22—a transmembrane glycoprotein and member of the SIGLEC family involved in the negative regulation of BCR signaling and maintaining B-cell tolerance [142,143]. It is highly expressed on normal as well as malignant B cells but is absent on other non-B-cell lineages and HSCs. CD22 is readily internalized after ADC binding, making it a feasible target for ADC-based therapies [28]. Commercially available is lnotuzumab ozogamicin (Besponsa), a calicheamicin immunoconjugate developed by Pfizer for the treatment of CD22-positive B-cell precursor acute lymphoblastic leukemia (B-ALL), which subsequently showed high efficiency against acute lymphoblastic leukemia (ALL) [63]. Inotuzumab ozogamicin is constructed from a humanized mAb targeting CD22 attached via an acid-cleavable linker to the cytotoxic N-acetyl-γ-calicheamicin with an average DAR of 5–7. In a phase 3 randomized, international, multicenter study (INO-VATE 1022), the safety and efficacy of the drug was evaluated against alternative chemotherapy approaches. The percentage of patients who met the primary endpoint (no evidence of disease and full recovery of blood counts after treatment) was twice as high in the Inotuzumab-ozogamicin-assigned group compared to reference treatment arms (35.8% vs. 17.4%, respectively) [28]. Furthermore, in June 2024, Inotuzumab ozogamicin received its first pediatric approval in the USA for patients aged ≥ 1 year suffering from B-ALL [144].

More recently, another CD22-targeted ADC, Moxetumomab pasudotox (Lumoxit), became the first immunotoxin to be globally approved for managing relapsed or refractory hairy cell leukemia (HCL) but was permanently withdrawn from the US (2023) and European (2021) markets following a decision by the manufacturer AstraZeneca.

CD79 is a heterodimeric transmembrane protein that takes part in the formation of the BCR complex and its downstream signaling following antigen recognition. CD79 consists of two immunoglobulin chains, CD79a and CD79b, paired by a disulfide bond. Cross-linking of the BCR by the binding of bivalent or multivalent antigens triggers a signal transduction cascade that can lead to apoptosis or promote cell division in response to rescue signals from T-cells [145]. Furthermore, the cross-linked BCR is transferred to lysosome-like compartments of the major histocompatibility complex class II as part of the antigen presentation by B-cells. This particular aspect in the biology of CD79 makes it a distinctly attractive target for ADCs, as endogenous CD79 trafficking would naturally deliver them to lysosomal compartments [146].

Certain oncogenic changes in the immunotyrosine-based activation motif (ITAM) of CD79a and CD79b have been reported to occur in diffuse large B-cell lymphoma (DLBCL), which drive the segregation of the activated B-cell-like subtype [147,148,149]. Of the two heterodimers, CD79b targeting has been demonstrated to achieve superior performance in ADC design, regardless of CD19 expression [150,151]. Studies have shown that anti-CD79b immunoconjugates with potent payloads like MMAF or DM-1 induce sustained tumor regression or complete remission in preclinical models, whereas ADCs targeting CD79a often result in tumor relapse [22]. In 2019, both the FDA and EMA granted approval for Polatuzumab vedotin, a CD79b-directed MMAE conjugate, for the treatment of DLBCL.

**BCMA**

The B-cell maturation antigen (BCMA) has emerged as a promising target for multiple myeloma (MM) therapies, being preferentially expressed by mature B lymphocytes and deficient in HSCs and nonhematopoietic tissue [152]. It is a member of the tumor necrosis factor receptor superfamily (TNFRSF17), which is selectively expressed during plasma cell differentiation and sustains the survival of long-lived plasma cells (PCs) [153,154]. BCMA cell survival signaling is mediated by the action of two key ligand molecules: the B-cell activating factor (BAFF) and the proliferation-inducing ligand (APRIL). BCMA overexpression and constitutive activation are identified as a pathogenetic mechanism in MM in both preclinical and clinical studies, serving both as a diagnostic marker and a therapeutic target in novel treatment approaches [152,154,155]. However, BCMA receptors can undergo γ-secretase-mediated shedding from B-cells’ surface, which may reduce their density on myeloma cells and compromise drug delivery [152]. The ADC Belantamab mafodotin (Blenrep) is a monoclonal antibody designed for targeted delivery of its antimitotic MMAF payload to BCMA+ MM cells. Belantamab mafodotin received accelerated market approval in the US and Europe in 2020 as a first-in-class medication but was later discontinued by the FDA based on clinical results from the phase III DREAMM-3 study failing to meet efficacy requirements [156,157].

**Molecular targets in Hodgkin’s lymphoma**

CD30 is another transmembrane glycoprotein belonging to the tumor necrosis factor receptor (TNFR) superfamily. It is expressed on a subset of activated B- and T-cells and is frequently found on various lymphoid neoplasms, including pathognomonic Reed–Sternberg cells of Hodgkin’s lymphoma [158]. The binding of CD30 by its CD30L promotes cell survival and proliferation by the recruitment of TNFR-associated factors (TRAFs) and their adaptor proteins, which activate downstream NF-kB and MAPK kinase signaling pathways. CD30 is upregulated in anaplastic large cell lymphoma (ALCL), cutaneous T-cell lymphoma (CTCL), as well as various Epstein–Barr virus (EBV)-driven lymphomas (classical HL, Burkitt’s lymphoma, and diffuse large B-cell lymphoma), but is relatively scarcely expressed in normal tissues, making it a lucrative target in ADC development [159,160,161,162]. Brentuximab vedotin is an anti-CD30 chimeric monoclonal antibody conjugated with the tubulin inhibitor MMAE via a protease-sensitive linker that has shown high responsiveness and manageable toxicity in the treatment of CD30+ lymphoproliferative disorders [162].

#### 4.1.2. Molecular Targets in Myeloproliferative Diseases

CD33 is the smallest member of the SIGLEC family and belongs to the differentiation antigens specifically expressed on stem cells of the myeloid lineage, including myeloblasts and monoblasts, monocytes/macrophages, granulocyte precursors, and mast cells; however, it is not present on normal CD34+ pluripotent HSCs or non-hematopoietic tissues [163,164,165]. Its downstream signaling is not entirely understood, although in vitro experiments suggest its inhibitory function on cellular activation and proliferation via the ITIM domain. Consistent with these findings, CD33 expression declines with the maturation of the myeloid lineage, restraining the proliferative capacity of peripheral granulocytes and tissue macrophages [165,166]. Naturally, about 90% of acute myeloid leukemia (AML) cases are CD33+, as defined by the abundant expression of the myeloid-specific antigen on more than 20–25% of immature leukemic blasts. This laid the molecular foundation for the design and development of the CD33-targeted ADC Gemtuzumab ozogamicin, readily taken up by myeloid blasts and facilitating the delivery of its genotoxic calicheamicin payload [167].

### 4.2. Molecular Targets of ADCs in Solid Tumor Malignancies

In contrast to oncohematological diseases, ADCs designed for the treatment of solid tumors must traverse tissue barriers, extravasate, and overcome the complexities of the tumor microenvironment (TME) to reach their target cells and exert their therapeutic effects [22,168]. In recent years, however, advanced ADC therapeutics have shifted focus towards difficult-to-treat solid tumor malignancies with unmet needs, leveraging new engineering technologies to develop fragment antibody frameworks with equally high binding affinities but improved tissue penetrability [19,169]. For example, antibody fragment–drug conjugates (FDCs) based on scFv (single-chain variable fragments) carry only the pharmacophore domains for surface recognition and are much smaller in size, exerting stronger vascular and tissue penetrability and lower immunogenicity. On the downside, however, depriving the antibody of its fragmented crystallizable (Fc) region strips away its immune effector functions, such as ADCC and CDC, compared to native full-length IgG scaffolds used as drug-delivery platforms [115,170,171,172,173].

ADCs in this oncological field are typically targeted towards a variety of antigens, including tumor-associated membrane glycoproteins and receptors primarily involved in signaling pathways promoting cancerogenesis in epithelial malignancies. Clinically exploited targets currently include HER2 (Human Epidermal Growth Factor Receptor 2), TROP2 (Trophoblast Antigen 2), Nectin-4, FRα (Folate Receptor Alpha), and TF (Tissue Factor) [22,27,168]. Clinically approved ADCs for the treatment of solid tumor malignancies are summarized in Table 3.

**The ErbB RTK family**

HER2 belongs to the human epidermal growth factor receptor (EGFR) or ErbB family represented by four structurally similar receptor tyrosine kinases: EGFR (ErbB1/HER1), ErbB2 (HER2/neu), ErbB3 (HER3), and ErbB4 (HER4). Ligand binding to their extracellular domains leads to the formation of functionally active homo- and heterodimers, activation of the tyrosine kinase domain, and receptor autophosphorylation at a number of tyrosine residues. Multiple adapter proteins and downstream signaling cascades, such as Ras/Raf/MAPK (mitogen-activated protein kinase) and PI3K/Akt pathways, are recruited, exerting regulatory effects on cell proliferation, differentiation, and survival. Constitutive hyperactivation of EGFR-signaling pathways as a result of gene amplification, gain-of-function point mutations, or increased expression is present in a number of malignancies of epithelial origin, including breast and ovarian carcinomas (Her2/neu proto-oncogene), lung cancer (ErbB1, ErbB3), head and neck cancer, colorectal cancer, glioblastomas, etc. [174,175,176,177,178,179].

ErbB2 is the only orphan receptor in the EGFR family for which no endogenous ligand has been found. This may be attributed to the unique structure of its extracellular domain, occupying a permanent open conformation unfit for ligand binding [180,181,182,183]. As a result, ErbB2 is the preferred dimerization partner for other ErbB receptors, enhancing their signaling and preventing their normal internalization and inactivation [184,185]. The gene for ErbB2 is amplified or overexpressed in approximately 30% of breast cancer carcinomas and in many other malignancies, including ovarian cancer, gastric cancer, bladder carcinoma, and non-small cell lung cancer (NSCLC). Being a common primary mechanism of solid tumor initiation, progression, and metastasis, targeting the ErbB2 receptor is an obvious and well-studied approach for selective tumor delivery of bioactive agents [186,187,188]. Several anti-HER2 monoclonal antibodies armed with antimitotic agents (DM1 and MMAE) or TOP I inhibitors (deruxtecan, Dxd) have asserted their place in the clinical management of the associated conditions. In addition, more sophisticated next-generation ADCs (i.e., Disitamab Vedotin and Trastuzumab deruxtecan) have demonstrated remarkable efficacy in clinical studies (C003 CANCER and DESTINY-Breast04, respectively) against heterogeneous tumors with varying or low levels of HER2 expression owing to their high loading capacity and pronounced secondary bystander effects on neighboring tumor cells [89,189,190,191].

**Figure 10:**
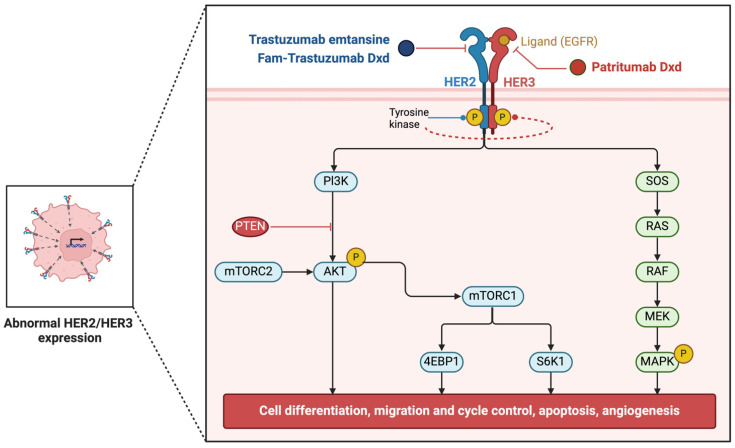
Sustained HER2/HER3 signaling in epithelial carcinomas as a resistance mechanism against HER2-targeting therapies. The tyrosine kinase domain of HER2 mediates the heterophosphorylation and activation of HER3 (dashed arrow), bypassing HER2 blockade. Dual HER2/HER3 inhibition may help overcome compensatory mechanisms of HER3 overexpression, leading to resistance in solid tumors. Created in BioRender. Mihaylova, R. (2024).

A newly explored target of the ErbB family is ErbB3, which has been found to be devoid of intrinsic autophosphorylation activity but to serve as an allosteric activator in formed heterodimers, particularly with ErbB2. Transphosphorylation of ErbB3 by its preferred partner ErbB2 triggers potent prosurvival signaling, making the ErbB2–ErbB3 complex the most oncogenic of the possible ErbB dimerization pairs [176,177,192]. HER3 is often overly co-expressed in HER2-positive breast cancer, as well as in other malignancies, including ovarian, prostate, lung, bladder, colorectal, and squamous cell carcinomas. What is more intriguing, recent studies have demonstrated that targeted inhibition of HER2 activity results in compensatory upregulation of HER3 transcription and phosphorylation, maintaining persistent PI3K/AKT signaling and eventually causing treatment failure (Figure 10) [176,193,194,195]. In view of this, Merck and Daiichi Sankyo jointly developed the first-in-class HER3-targeted DXd antibody–drug conjugate patritumab deruxtecan, which in the HERTHENA-Lung02 phase 3 trial demonstrated statistically significant improvement in progression-free survival vs. platinum-based chemotherapy in patients with locally advanced or metastatic EGFR-mutated NSCLC. Patritumab deruxtecan was granted a Breakthrough Therapy Designation in 2021 and Priority Review to the Biologics License Application (BLA) in 2023 and awaits its FDA approval as a third-line treatment for patients with EGFR-mutated NSCLC [196,197].

**TROP-2**

TROP-2 (Trophoblast Antigen 2), also known as tumor-associated calcium signal transducer 2, is another surface glycoprotein showing distinctly high expression profiles in various solid tumor diseases, including breast, prostate, colorectal, gastric, pancreatic, and lung cancers, but limited expression in normal human tissues. Although there is no evidence of oncogenic TROP-2 mutations at the genetic level, it has been suggested that its expression and activity are disinhibited by epigenetic modifications resulting in epithelial–mesenchymal transition (EMT), particularly in breast and prostate carcinomas [198,199,200,201,202].

TROP-2 contains extracellular (ECD) and intracellular (ICD) domains engaging in both regulatory and signaling functions, which promote cell survival and proliferation and modulate cell motility, cell–cell adhesion, and communication processes. In tumors, TROP-2 has been shown to undergo regulated intramembrane proteolysis (RIP) by the TNFα-converting enzyme (TACE), shedding its ECD and serving as an oncogenic switch for tumor initiation and progression. This enables the translocation of the cleaved ICD to the nucleus, where it acts as a transcription factor and induces the expression of proto-oncogenes (i.e., c-myc) and cell cycle-accelerating regulatory proteins (cyclin D1). ICD also activates the phospholipase C cascade, which raises intracellular Ca^2+^ and thus activates the prosurvival MAPK/ERK signaling pathway. The ECD of Trop2 is involved in cell adhesion and migration processes through the integrin β1-RACK1-FAK-Src signaling axis and can promote cancer cell invasion and metastasis [25,198,199,203,204,205].

Among the epithelial malignancies, the highest expression of Trop-2 is found in triple-negative breast cancer (TNBC); however, heterogeneous activation is still encountered across all breast cancer subtypes. In addition to HER2, the identification of Trop-2 as a therapeutic target opened the door to a new paradigm of ADC-based antitumor approaches, with sacituzumab govitecan being the first to be FDA-approved. Sacituzumab govitecan is a humanized anti-Trop-2 monoclonal antibody conjugated with the topoisomerase I inhibitor SN-38, which has shown durable antitumor responses and survival benefits in high-risk patients with both metastatic TNBC and hormone-receptor-positive breast cancer. Other Trop-2-directed therapies are also under development and clinical investigation [84,86,205,206].

**Nectin-4**

Nectin-4 (Poliovirus Receptor-Related Protein 4) is another emerging molecular target in ADC design, implicated in the onset and development of various epithelial cancers, including breast, urothelial, and lung carcinomas [207]. It is a transmembrane protein within the nectin family of immunoglobulin-like cell adhesion molecules (ICAM) governing calcium-independent junctional as well as cytoskeletal rearrangements and cell–cell communications [208]. In contrast to constitutive Nectins 1–3, Nectin-4 is normally not present in healthy adult tissues, and its expression is predominantly confined to embryonic and placental tissues. Its amplification and overexpression in solid tumors trigger epithelial–mesenchymal transition and promote tumor angiogenesis, proliferation, and metastasis [207,209]. Furthermore, under hypoxic conditions, nectin-4 is subjected to ectodomain shedding via proteolytic cleavage by cell surface metalloproteinases. Its soluble form has been shown to interact with integrin-β4 on endothelial cells, facilitating angiogenesis through Src, PI3K, Akt, and NO signaling pathways [207,210]. Plasma levels of cleaved Nectin-4 have also been found to be of prognostic value in various cancers, predicting poor progression-free survival, particularly in TNBC [211]. In 2019, the first MMAE-conjugated ADC platform targeting Nectin-4 (Enfortumab vedotin) received accelerated approval by the FDA as a next-line treatment option for locally advanced and metastatic urothelial carcinoma unresponsive to anti-PD-1/L1 therapy and platinum-based chemotherapy [209,212].

**Tissue Factor (TF)**

The crosslink between the hemostatic system and cancer is now well established, with tissue factor (TF) recognized as their intersection point [213]. TF, also known as thromboplastin Factor III or CD142, is a transmembrane glycoprotein serving as a cofactor for the circulating zymogen FVII and a major initiator of the extrinsic blood coagulation cascade [214,215]. TF is constitutively expressed in various tissues, including subendothelial spaces and adventitia, where it acts as a hemostatic envelope in concert with calcium, phospholipids, and other coagulation factors to halt bleeding. Naturally, TF is abundantly expressed in highly vascularized organs, like the brain, kidneys, lungs, heart, and placenta, exerting potent pro-coagulant activity [216]. However, various pathological conditions, including cancer and inflammation, cause a marked shift in TF expression and activity in response to hypoxia, propagating its paracrine procoagulant and proangiogenic signaling [215,217]. The TF:FVIIa complex induces proteolytic cleavage of protease-activated G-protein-coupled receptors (PARs), which in turn facilitate TF shedding and its secretion into the circulation in the form of extracellular vesicles (TF+EVs) [216,217]. Beyond their prothrombotic effects, TF+EVs trigger the transactivation of integrins and multiple RTKs, including EGFR, PDGFRβ, and IGF-1R on remote cells, promoting cell division, angiogenesis, metastasis, and cell transition to a cancerous stem-like state [213,216,218].

TF is already a clinically well-validated target in a broad range of solid tumors, including ovarian and cervical cancers, NSCLC, colorectal cancer, pancreatic cancer, and prostate carcinomas, where its prevalent expression has been associated with poor prognosis [219,220,221]. The constant and high turnover of TF, even on resting tumor cells, enables efficient intracellular delivery of ADC-based cancer therapeutics [221]. Tisotumab vedotin was the first-in-class MMAE-conjugated anti-TF monoclonal antibody licensed in the US (2021) and Europe (2024) as a second- or third-line treatment for recurrent cervical cancer. In the global, randomized, open-label phase 3 study InnovaTV 301, Tisotumab vedotin immunotherapy resulted in a 30% lower risk of death compared to conventional chemotherapy [222,223].

**Folate Receptor Alpha (FRα)**

Folate Receptor Alpha (FRα) is a membrane-bound receptor involved in the cellular uptake of folic acid. Its function and role in physiological and pathological conditions were discovered decades ago, along with drugs that target intracellular folate homeostasis, such as methotrexate and pemetrexed [224]. FRα is a glycosyl-phosphatidylinositol (GPI) anchor glycoprotein with a higher affinity for oxidized than reduced folate forms, accommodating their transport to the cytoplasm via potocytosis [225]. Folic acid or vitamin B9 performs its main functions in its reduced state, tetrahydrofolic acid, serving as an acceptor and donator of one-carbon residues in DNA synthesis, particularly in the methylation of uracil to thymine [226]. Being essential for replication, folate utilization is intensified in various cancers, i.e., ovarian, breast, and lung carcinomas, by the overexpression of the FRα receptor [227,228]. Additionally, the FRα elicits pro-growth intracellular responses independent of one-carbon metabolism by phosphorylation and activation of the survival JAK/STAT3 and ERK1/2 signaling pathways [224,228,229,230]. However, the clinical efficacy of FRα-targeting ADCs may be limited by the pronounced receptor’s shedding into circulation, particularly observed in patients with ovarian cancers [231]. Nevertheless, the first-in-class DM4-conjugated ADC, Mirvetuximab soravtansine, proved superior to chemotherapy with respect to progression-free and overall survival and objective remission rate in FRα-positive platinum-resistant ovarian cancer. Based on these encouraging Phase 3 results, the FDA granted accelerated approval for Mirvetuximab soravtansine as a second to fourth-line treatment for FRα+, platinum-resistant epithelial ovarian, fallopian tube, and primary peritoneal cancer in 2022 [232].

**c-MET**

c-MET is a receptor tyrosine kinase belonging to the MET (MNNG HOS transforming gene) family, playing a vital role in physiological processes such as embryogenesis, wound healing, and post-injury responses [233]. The endogenous ligand for c-MET is the hepatocyte growth factor (HGF), whose binding stimulates a wide network of prosurvival downstream pathways, including PI3K/AKT, Ras/MAPK, JAK/STAT, SRC, and Wnt/β-catenin [234,235]. Aberrant activation of the c-Met axis due to genomic amplification, mutation, overexpression, or alternative splicing sustains a pro-oncogenic phenotype in various solid tumors, such as NSCLC, gastrointestinal, and hepatocellular carcinomas. Several small tyrosine kinase inhibitors targeting c-MET have been evaluated in clinical trials, with results ranging from relatively high response rates to prominent failure [233]. The first attempts to develop an ADC-based c-MET-targeted therapy were also not far behind. Telisotuzumab vedotin (Teliso-V) is a c-Met-directed immunoconjugate with an MMAE cytotoxic payload, which produced durable responses as monotherapy in c-MET-overexpressing NSCLC with a wildtype EGFR gene. The drug is currently undergoing several clinical trials (NCT05513703, NCT04928846) in patients with advanced/metastatic NSCLC [236,237,238].

## 5. Future Perspectives in ADC Design

The evolution of ADCs as a drug delivery platform is primarily driven by the molecular advances made in tumor pathology, as well as the accumulated clinical experience with commercially available immunotherapies, unveiling their challenges and limitations. As mentioned, small-format “biologics” such as scFv, Fab, designed ankyrin-repeat proteins (DARPins), single-domain antibodies (VHHs), and nanobodies are coming more and more into focus, dramatically changing the distributional pattern, scope of target antigen recognition, and loading capacity of the resultant ADC construct [115,239,240]. Single-domain antibodies, also referred to as heavy-chain-only antibodies (HCAbs), are naturally occurring antibodies devoid of light chains and CH1 domains first found in immunized animals from the Camelidae family. Given this, their size amounts to about half that of conventional immunoglobulins (ca. 75 kDa), yet they retain extremely high selectivity and binding affinity via the terminal variable domains. While the smaller format can enhance tissue penetration, it does not necessarily resolve the inherent limitations of conventional mAbs, such as stability, solubility, and tendency toward aggregation. A further simplification of the scaffold can be achieved using synthetic, naïve, or immune libraries to produce the smallest functional antibody domains, called nanobodies (Nbs) [241,242,243]. Featuring a much more compact peptide structure of 2–17 kDa, Nbs exert a much higher tissue permeability and low aggregation rate, which allows their heavy loading with cytotoxins making up to 30% of the overall conjugate mass compared to 2–3% for a typical IgG [244]. Furthermore, the Nbs framework is distinguished by extreme chemical, pH, and thermal stability, including in the presence of proteases, suggesting the potential for alternative routes of administration, such as oral delivery [241]. Variable VH and VL domains of naked Nbs can also be genetically fused via a peptide linker into small single-chain variable fragments (scFvs) or used as a targeting tool on liposomes or other nanodelivery platforms [242]. The smaller ADC formats of these classes may also provide access to molecular targets located behind the blood–brain barrier or intracellular targets of the tumor antigen repertoire, aiding the effector functions of the payload agents [241,242,245]. Based on the screening of large-scale libraries, the spectrum of potential payload candidates is also expanding, spanning over extravagant immunotoxins (PE-38), radionuclides, oligonucleotides (siRNA), and photosensitizers (IRDye/700DX) [245,246]. For instance, mirzotamab clezutoclax is an ADC under clinical investigation employing the B7-H3 checkpoint molecule for the targeted delivery of a proapoptotic BCL-XL inhibitor with potent boosting effects on antitumor immunity [28]. Dual antigen targeting (i.e., HER2/HER3 or CD3/CD28), as well as dual payload approaches, may help overcome resistance issues related to ADC internalization or inadequacy in antitumor humoral or single-agent responses [10,28,240,247]. For example, ADCs targeting both DNA and microtubule dynamics have demonstrated a more durable response in HER2-positive breast cancer models, allowing dose reduction of individual agents with synergistic activities, bypassing resistance mechanisms, and avoiding overlapping toxicities [33,90]. A dual-payload ADC construct targeting CD276/B7-H3 and microtubule polymerization has been demonstrated to effectively inhibit cell proliferation and recruit immune cells in both mouse and patient-derived xenograft TNBC models [248]. Another dual-payload conjugate of Trastuzumab (Tmab-VcMMAE-SMCC-DM1) carrying both MMAE and DM1 has shown promising in vitro activity against HER2+ breast and colon cancer cell lines, superior to the reference Tmab drug [249]. An anti-HER2 ADC containing both MMAE and MMAF has shown remarkable therapeutic efficacy in two mouse models of refractory breast cancer with heterogeneous HER2 expression [247]. Currently, several dual-payload ADCs are in the process of preclinical evaluation, exhibiting enhanced anticancer efficacy in cellular and animal models. However, their clinical validation remains problematic due to insufficient payload synergism and/or the complexity of their design and synthesis related to payloads, linker and mAb types, and conjugation approaches [250].

Another recent and extravagant approach to overcome resistance issues with immunotherapy is to activate the innate antitumor response, tampering with both the type of antibody framework (e.g., bi- or multispecific mAbs) and the type of payload used. A novel class of unconventional ADC formats explores the immunostimulatory effects of agents like Toll-like receptor 7 (TLR7) and stimulator of interferon genes (STING) agonists, which drive antitumor immunity by promoting antigen presentation and direct killing of cancer cells, particularly in immunologically “cold” tumors with low antigen expression [251,252,253]. Co-delivery of STING and TLR7 stimulatory ligands results in a vigorous activation and maturation of dendritic cells in a synergistic manner, increased expression of T-cell co-stimulatory molecules (CD80 and CD86), initiation of the adaptive immune response, and modulation of immune cell function via cytokine secretion [254]. Advantageous therapeutic results have already been reported for several STING/TLR7-based ADCs with potent immune-boosting properties, particularly for the treatment of colorectal cancer, multiple myeloma, and HER2-expressing tumors [251,254,255]. Another emerging new modality is the degrader–antibody conjugate (DAC), which targets protein degradation in tumor cells with specific antigen characteristics using Proteolysis-Targeting Chimeras (PROTACs). This new technology relies on the recognition and effector functions of bifunctional PROTAC payloads composed of a ligand against a protein of interest (POI) linked to an E3 ligase recruiter via a chemical linker [256,257]. PROTACs have been shown to exert a broad spectrum of protein-degrading activities on cancer-related targets, including androgen receptors, estrogen receptors, folate receptors, and various kinases; however, they tend to lack tissue specificity as E3 ligases are ubiquitously expressed, which promotes the development of DACs to achieve selective tumor internalization and lysosomal release of protein degraders using antibodies as a mechanism for active tumor targeting. Several working groups have already reported on the design, synthesis, and preclinical efficacy of DAC entities targeting the HER2 receptor, but their clinical evaluation is still underway [256,257,258,259,260,261,262,263,264].

## 6. Conclusions

Despite the prevailing tendency towards innovative, highly specific, immunoconjugate drug delivery systems, their real impact on the overall process of diagnosis, treatment, and follow-up of oncological diseases in healthcare systems remains very limited. This has less to do with the therapeutic potential of this approach per se, but rather, is related to breaking new ground in the field of pharmaceutical and biomedical sciences. With only three decades of actual research on the subject, many of the key fundamental and applied aspects of these innovative biotechnologies are not yet fully understood, and their costs remain prohibitive. Undoubtedly, the identification of key biological markers that allow precise targeting of malignantly transformed cells, as well as the elaboration of new drug candidates with optimized safety and efficacy profiles, is an integral part of future studies of next-generation ADCs. Ultimately, the practical application of ADCs will advance in parallel with our growing knowledge and expertise of disease processes at the molecular level, where the successful identification and validation of appropriate targets will open avenues for the development and implementation of ever more sophisticated therapeutic modalities.

## Figures and Tables

**Table 1:** The most common immunophenotype characteristics of hematopoietic and lymphoid neoplasms according to their lineage origin [29,134,137,138,139,140].

Malignant Disease	Immunophenotype
NHL (B-ALL)	CD19, CD20, CD22, CD79
MM	BCMA
HL	CD30, CD25, GD197
AML	CD33, CD25, CD64, c-Kit, FLT3
T-ALL	CD2, CD3, CD4, CD5, CD7

NHL: Non-Hodgkin’s lymphoma; B-ALL: B-cell acute lymphoblastic leukemia; MM: multiple myeloma; HL: Hodgkin’s lymphoma; AML: acute myeloid leukemia; T-ALL: T-cell acute lymphoblastic leukemia.

**Table 2:** Clinically approved ADCs for the treatment of hematological malignancies [2,8,27,28,29,33,115].

Drugs	Target	Linker	Payload	DAR	Indications	Approval
**Gemtuzumab ozogamicin** Mylotarg^®^	CD33	hydrazone	Calicheamicin	2–3	Acute myeloid leukemia	2000 (FDA) 2017 (EMA)
**Brentuximab vedotin** Adcentris^®^	CD30	mc-VC-PABC	MMAE	4	Mesenchymal large cell lymphoma	2011 (FDA) 2012 (EMA)
**Inotuzumab ozogamicin** Besponsa^®^	CD22	hydrazone	Calicheamicin	2–3	Acute lymphoblastic leukemia	2017 (FDA) 2017 (EMA)
**Moxetumomab pasudotox** Lumoxit^®^	CD22	mc-VC-PABC	PE38	NA	Relapsed or refractory HCL	2018 (FDA) *
**Polatuzumab vedotin** Polivy^®^	CD79b	mc-VC-PABC	MMAE	3.5	Diffuse large B-cell lymphoma	2019 (FDA) 2020 (EMA)
**Belantamab mafodotin** Blenrep^®^	BCMA	mc	MMAE	4	Multiple myeloma	2020 (FDA) ** 2020 (EMA)
**Loncastuximab tesirine** Zynlonta^®^	CD19	dipeptide	PBD	2.3	Relapsed/refractory diffuse large B-cell lymphoma	2021 (FDA)

* discontinued as of 2024; ** discontinued in 2023.

**Table 3:** Clinically approved ADCs for the treatment of solid tumor malignancies [2,8,28,29,33,115].

Drugs	Target	Linker	Payload	DAR	Indications	Approval
**Trastuzumab emtansine** Kadcyla^®^	HER2	SMCC	DM1	3.5	HER2-positive breast cancer	2013 (FDA) 2013 (EMA)
**Enfortumab vedotin** Padcev^®^	Nectin-4	mc-VC-PABC	MMAE	3.8	Uroepithelial tumor	2019 (FDA) 2024 (EMA)
**Fam-Trastuzumab Dxd** Enhertu^®^	HER2	tetrapeptide	Deruxtecan	8	HER2+/low breast cancer	2019 (FDA) 2019 (EMA)
**Sacituzumab govitecan** Trodelvy^®^	TROP2	CL2A	SN38	7.6	Triple-negative breast cancer	2020 (FDA) 2021 (EMA)
**Cetuximab sarotalocan** Akalux^®^	EGFR	NA	IRDye/700DX	1.3–3.8	Head and Neck Tumors	2020 (Japan)
**Disitamab vedotin** Aidixi^®^	HER2	mc-VC-PABC	MMAE	4	HER2+ gastric cancer	2021 (China)
**Tisotumab vedotin** Tivdak^®^	TF	mc-VC-PABC	MMAE	4	Recurrent or metastatic cervical cancer	2021 (FDA)
**Mirvetuximab soravtansine** Elahere^®^	FRα	Sulfo-SPDB	DM4	3.4	FRα+ drug-resistant epithelial, ovarian, fallopian tube, or primary peritoneal cancer	2022 (FDA)
**Patritumab Dxd**	HER3	tetrapeptide	Deruxtecan	8	NSCLC	2023 (FDA) * 2024 (EMA)
**Telisotuzumab vedotin** Teliso-V^®^	C-MET	mc-VC-PABC	MMAE	3.1.	NSCLC	2021 (FDA) **

* issued a complete response letter for the biologics license application; ** granted breakthrough therapy designation.
